# Comparison of Biometric Measurements Between IOL Master 700 and MS-39 in Post-LASIK Myopic Eyes: Implications for IOL Selection in Cataract Surgery

**DOI:** 10.1055/a-2763-7114

**Published:** 2026-02-23

**Authors:** Giacomo Edoardo Bravetti, Giorgio Enrico Bravetti, Emmanouil Blavakis, Giacomo Savini, Gian Maria Cavallini

**Affiliations:** 1 Ophthalmology Department, University Hospital Modena, Italy; 2 Ophthalmology Department, Geneva University Hospitals, Geneva, Switzerland; 3 Ophthalmology Department, G. B. Bietti Foundation I. R. C. C. S., Rome, Italy

**Keywords:** IOL, myopia, LASIK, biometry, cataract surgery, refractive Surgery, Myopie, LASIK, Augenbiometrie, refraktive Chirurgie, Kataraktoperation, Intraokularlinse

## Abstract

**Background** Accurate ocular biometry is essential for achieving optimal refractive outcomes in cataract surgery. In patients with prior myopic LASIK, however, biometric calculations remain particularly challenging due to altered corneal anatomy.

**Purpose** This study aims to compare preoperative biometric measurements obtained from the Zeiss IOL Master 700 and the CSO Ray-Tracing MS-39 in patients with a history of myopic LASIK. The goal is to assess the consistency and clinical relevance of these measurements to support intraocular lens (IOL) power selection in this complex patient group.

**Setting** Longitudinal, monocentric, retrospective study, conducted at the Ophthalmology Department of the University Hospital of Modena.

**Methods** Patients over 18 years of age scheduled for cataract surgery with a history of myopic LASIK and available preoperative biometry from both the IOL Master 700 and MS-39 were included. Exclusion criteria were incomplete clinical records, prior intraocular surgery, or significant ocular pathologies affecting measurement accuracy. The primary outcomes were differences in calculated emmetropic IOL power and predicted postoperative refraction. Secondary outcomes included comparisons of anterior chamber depth (ACD), central corneal thickness (CCT), average keratometry (K-avg), total corneal power (total keratometry [TK] from IOL Master 700 vs. mean pupil power [MMP] from MS-39), and predicted post-operative spherical equivalent (SE) by using the same IOL power, calculated from the IOL Master 700.

**Results** Seventy-six eyes from 41 patients (mean age 46.54 ± 12.70 years; 60.98% female) were analysed. The average axial length was 25.93 ± 1.55 mm. An IOL constant of 119.0 was used uniformly across both devices. Statistically significant differences were found in all biometric parameters: ACD (p = 0.00 001), CCT (p = 0.0189), K-avg (p = 0.00 009), and total keratometry (p = 0.00 001). However, no significant differences were observed in the selected IOL power for emmetropia (p = 0.72 527), the predicted postoperative SE of the IOL Master 700 vs. the MS-39 (p = 0.81 642), or the predicted postoperative SE by using the same IOL power, calculated from the IOL Master 700, (p = 0.38 938).

**Conclusions** Despite statistically significant differences in individual biometric parameters between the IOL Master 700 and MS-39, these did not impact final IOL power selection or predicted refraction in post-myopic LASIK patients. Utilizing multiple biometry devices may still enhance confidence and accuracy in surgical planning for this complex group of patients.

## Zusammenfassung

**Hintergrund** Eine präzise okuläre Biometrie ist entscheidend, um optimale refraktive Ergebnisse bei der Kataraktchirurgie zu erzielen. Bei Patienten mit vorangegangener myoper LASIK bleiben biometrische Berechnungen jedoch aufgrund der veränderten Hornhautanatomie besonders schwierig.

**Zielsetzung** Ziel dieser Studie ist der Vergleich präoperativer biometrischer Messungen, die mit dem Zeiss IOL Master 700 und dem CSO Ray-Tracing MS-39 bei Patienten mit anamnestisch bekannter myoper LASIK gewonnen wurden. Es soll die Übereinstimmung und klinische Relevanz dieser Messungen zur Unterstützung der Auswahl der Intraokularlinsen-(IOL-)Stärke in dieser komplexen Patientengruppe bewertet werden.

**Setting** Longitudinale, monozentrische, retrospektive Studie, durchgeführt an der Augenklinik des Universitätsklinikums Modena.

**Methoden** Eingeschlossen wurden Patienten über 18 Jahre, die für eine Kataraktoperation geplant waren, eine Vorgeschichte myoper LASIK aufwiesen und bei denen präoperative Biometriedaten sowohl vom IOL Master 700 als auch vom MS-39 vorlagen. Ausschlusskriterien waren unvollständige klinische Daten, vorausgegangene intraokulare Operationen oder signifikante okuläre Pathologien, die die Messgenauigkeit beeinflussen. Primäre Endpunkte waren Unterschiede in der berechneten emmetropen IOL-Stärke und der vorhergesagten postoperativen Refraktion. Sekundäre Endpunkte umfassten Vergleiche der Vorderkammertiefe (ACD), der zentralen Hornhautdicke (CCT), der mittleren Keratometrie (K-avg), der gesamten Hornhautbrechkraft (totale Keratometrie [TK] des IOL Master 700 vs. mean pupil power [MMP] des MS-39) sowie des vorhergesagten postoperativen sphärischen Äquivalents (SE) unter Verwendung derselben IOL-Stärke, berechnet mit dem IOL Master 700.

**Ergebnisse** Sechsundsiebzig Augen von 41 Patienten (mittleres Alter 46,54 ± 12,70 Jahre; 60,98% weiblich) wurden analysiert. Die durchschnittliche axiale Länge betrug 25,93 ± 1,55 mm. Für beide Geräte wurde einheitlich eine IOL-Konstante von 119,0 verwendet. In allen biometrischen Parametern zeigten sich statistisch signifikante Unterschiede: ACD (p = 0,00001), CCT (p = 0,0189), K-avg (p = 0,00009) und totale Keratometrie (p = 0,00001). Es fanden sich jedoch keine signifikanten Unterschiede hinsichtlich der gewählten IOL-Stärke für Emmetropie (p = 0,72527), des vorhergesagten postoperativen SE (IOL Master 700 vs. MS-39; p = 0,81642) oder des vorhergesagten postoperativen SE bei Verwendung derselben IOL-Stärke, berechnet mit dem IOL Master 700 (p = 0,38938).

**Schlussfolgerungen** Trotz statistisch signifikanter Unterschiede in einzelnen biometrischen Parametern zwischen dem IOL Master 700 und dem MS-39 hatten diese keinen Einfluss auf die endgültige Auswahl der IOL-Stärke oder die vorhergesagte Refraktion bei Patienten nach myoper LASIK. Die Nutzung mehrerer Biometriegeräte kann dennoch das Vertrauen in die chirurgische Planung und deren Genauigkeit bei dieser komplexen Patientengruppe erhöhen.

## Introduction

Accurate ocular biometry remains the cornerstone of successful cataract surgery and postoperative refractive predictability. In recent decades, the increasing number of patients who have undergone corneal refractive surgery, particularly laser-assisted in situ keratomileusis (LASIK), has introduced significant challenges in intraocular lens (IOL) power calculation. The corneal remodelling induced by myopic LASIK alters the anterior-to-posterior fixed curvature ratio and invalidates the standard keratometric index (1.3375), leading to systematic errors in corneal power estimation and, consequently, refractive surprises after cataract surgery 1 , 2 , 3 .

Traditional biometers, such as partial coherence interferometry and early optical biometers, rely on assumptions about corneal geometry that do not hold true in post-refractive eyes. These devices typically estimate total corneal power based only on anterior curvature and a fixed refractive index, neglecting posterior curvature and corneal thickness variations 4 . With the advent of swept-source optical coherence tomography (SS-OCT) and anterior segment tomographers, more sophisticated technologies now allow for direct/indirect or ray-traced measurement of both anterior and posterior corneal surfaces. For instance, the Zeiss IOL Master 700 measures the anterior corneal curvature directly and estimates the posterior surface indirectly from the corneal pachymetry measurement 5 . In contrast, the CSO Ray-Tracing MS-39 provides direct measurements of both anterior and posterior corneal surfaces using ray-tracing technology, offering potentially more reliable IOL power estimations, especially in altered corneas 6 , 7 .

The Zeiss IOL Master 700 employs SS-OCT to provide “total keratometry” (TK), integrating anterior and posterior curvature data to refine corneal power estimation 8 . Conversely, the CSO Ray-Tracing MS-39 combines Placido-disc topography with high-resolution Scheimpflug tomography, enabling ray-tracing analysis of corneal power through “mean pupil power” (MPP). Both devices represent state-of-the-art approaches for biometry in complex eyes, yet the extent to which their measurements and IOL power predictions align remains incompletely characterized, particularly in patients with prior myopic LASIK.

The present study aims to compare preoperative biometric measurements obtained with the IOL Master 700 and MS-39 in post-myopic LASIK eyes undergoing cataract surgery. By analysing key biometric parameters, and predicted refraction, this work seeks to assess the consistency and clinical relevance of these technologies. Understanding the agreement and potential discrepancies between the two systems will help cataract surgeons in selecting the most appropriate measurement strategy and enhance confidence in IOL selection for this challenging patient population. To the best of our knowledge, this is the first comparative analysis of these two biometers for IOL power calculation in eyes with previous history of myopic LASIK.

## Methods

### Study design

The present study was a longitudinal, monocentric, retrospective study, conducted at a tertiary university centre (University of Modena and Reggio Emilia, Modena, Italy). Written informed consent was obtained from all included patients. The study complies with the tenets of the Declaration of Helsinki and was approved by the local ethical committee.

### Study population

Patients over 18 years of age scheduled for cataract surgery with a history of myopic LASIK and available preoperative biometry from both the IOL Master 700 and MS-39, between January 2019 and February 2025, were included. Exclusion criteria were incomplete clinical records, prior intraocular surgery, or significant ocular pathologies affecting measurement accuracy. An IOL constant of 119.0 was used uniformly across both devices.

### Baseline measurements

For each included patient, demographic and clinical data were recorded, including age, gender, and ocular history, with specific attention to prior myopic LASIK parameters. Preoperative ophthalmic assessment included best-corrected visual acuity (BCVA), manifest refraction, and slit-lamp biomicroscopy. Biometric data were obtained using both the IOL Master 700 (Carl Zeiss Meditec AG, Germany) and the MS-39 (CSO, Florence, Italy) devices. The following parameters were collected for each eye: axial length (AL), anterior chamber depth (ACD), central corneal thickness (CCT), average keratometry (K-avg), and total corneal power (total keratometry [TK] from IOL Master 700 vs. mean pupil power [MPP] from MS-39). All measurements were performed by experienced technicians under standardized lighting and fixation conditions. When clinically indicated, a spectral-domain OCT (Spectralis OCT, Heidelberg Engineering AG, Germany) and ultra-widefield fundus imaging (Optos California, Nikon Co. Ltd., Japan) were performed to evaluate the posterior segment status and exclude retinal pathology that could affect surgical planning or postoperative visual outcomes.

### Outcomes measures

The primary endpoints were the difference in calculated emmetropic IOL power and the difference in predicted postoperative SE obtained from the IOL Master 700 (using the Haigis-TK formula, which refers to a version of the Haigis intraocular IOL power calculation formula that incorporates TK measurements) vs. the MS-39 (using the CSO ray-tracing algorithm). Secondary outcomes included comparisons of key biometric parameters between the two devices: ACD, CCT, K-avg, total corneal power (TK from IOL Master 700 vs. MMP from MS-39), and predicted postoperative SE by using the same IOL power, calculated from the IOL Master 700.

### Statistical analysis

Descriptive statistics included mean and standard deviation (SD) for normally distributed variables, and median and interquartile range (IQR) for non-normally distributed variables. All tests were two-tailed and a P-value less than 0.05 was considered statistically significant. Statistical analyses were performed with commercially available software (Stata version 13.1; StataCorp, College Station, TX).

## Results

### Baseline characteristics of study population

Seventy-six eyes from 41 patients (mean age 46.54 ± 12.70 years; 60.98% were female; 52.63% were left eyes) were analysed. The average axial length was 25.93 ± 1.55 mm.

### Primary outcomes: calculated emmetropic IOL power and predicted postoperative refraction

No statistically significant differences were observed between the IOL Master 700 and the MS-39 in the IOL power required to achieve emmetropia (20.20 ± 3.26 D vs. 20.16 ± 3.09 D respectively, p = 0.72527) or in the predicted postoperative SE (− 0.001 ± 0.10 D vs. − 0.005 ± 0.10 D respectively, p = 0.81642). Primary outcomes are summarised in Table 1 .

**Table:** **Table 1** Primary outcomes.

	IOL Master 700	MS-39	t-test*
Emmetropic IOL Power (mean ± SD)	20.20 ± 3.26 D	20.16 ± 3.09 D	p = 0.72527
Predicted SE (mean ± SD)	− 0.001 ± 0.10 D	− 0.005 ± 0.10 D	p = 0.81642
D = dioptres, SD = standard deviation; * paired Studentʼs t-test			

### Secondary outcomes: biometric parameters

Statistically significant differences were observed in all biometric parameters: ACD (p = 0.00001), CCT (p = 0.0189), K-avg (p = 0.00009), and total corneal power (p = 0.00001). When the same IOL power (calculated by the IOL Master 700) was applied across both platforms, the predicted postoperative SE difference did not reach statistical significance (p = 0.38938). Secondary outcomes are summarised in Table 2 .

**Table:** **Table 2** Secondary outcomes.

	IOL Master 700	MS-39	t-test*
ACD (mean ± SD)	3.50 ± 0.38 mm	3.61 ± 0.37 mm	p = 0.00001
CCT (mean ± SD)	477.04 ± 37.69 µm	479.34 ± 36.97 µm	p = 0.0189
K-avg (mean ± SD)	39.66 ± 1.69 D	39.82 ± 1.61 D	p = 0.00009
Total corneal power (mean ± SD)	39.17 ± 1.81 D	38.43 ± 1.96 D	p = 0.00001
D = dioptres, SD = standard deviation, ACD = anterior chamber depth, CCT = central corneal thickness, k-avg = average keratometry; * paired Studentʼs t-test			

## Discussion

The increasing global prevalence of myopia, projected to affect approximately half of the worldʼs population by 2050 with about 10% having high myopia, coupled with the widespread adoption of corneal refractive procedures, has created a rapidly growing population of patients requiring cataract surgery after previous refractive interventions 9 , 10 . Cataract surgery in such patients demands additional preoperative considerations due to the altered corneal shape and the accuracy of preoperative measurements is essential in achieving the desired refractive outcomes 11 . The present study compared preoperative biometric measurements obtained from the Zeiss IOL Master 700 and the CSO Ray-Tracing MS-39 and used for IOL power calculation in patients with a history of myopic LASIK, providing valuable insight on the reliability of these two biometric devices.

Traditional biometers, estimate corneal curvature solely from anterior surface measurements, assuming a fixed physiological relationship between anterior and posterior corneal surfaces, a relationship that is altered in patients who have undergone refractive surgery 12 . Relying on this estimation can lead to inaccurate corneal curvature results in post-refractive surgery eyes. 13 Recent technological advancements have allowed for direct (e.g. MS-39) or indirect (e.g. IOL Master 700) measurement of the posterior corneal curvature, enabling the calculation of total keratometry (TK for IOL Master 700 and MMP for the MS-39), by taking into account both corneal surfaces. The accuracy of IOL power calculation using TK data has been demonstrated to be reliable in both non-operated and post-refractive surgery eyes 14 . Additionally, the high repeatability of both IOL Master 700 and MS-39 has been confirmed in previous studies 15 , 16 .

A study by De Rosa et al. comparing measured versus predicted posterior corneal astigmatism demonstrated better accuracy in IOL power calculations when measured posterior corneal astigmatism was used 17 . This could support the hypothesis that the MS-39 may be more accurate than IOL Master 700 in IOL power calculation. However, this study showed that both devices had comparable results in IOL power selection and predicted postoperative refraction in post-myopic LASIK patients, despite variations in individual biometric parameters.

Our findings suggest that although the two devices differ in the measurements of ACD, CCT, K-avg and total corneal power, these discrepancies did not translate into clinically significant differences in IOL power calculation for emmetropia. However, we noted an average difference of 0.11 mm on ACD and 0.74 D on total corneal power, with smaller but statistically significant differences in CCT and K-avg. Therefore, caution is advised when using these values in various IOL power calculation formulas, including online calculators.

Despite the large number of formulas available, most are unable to provide consistent and precise postoperative refractive outcomes in eyes with a history of corneal refractive surgery, with over 30% of eyes showing refractive prediction errors greater than 0.50 D 18 . One major reason is that following corneal refractive surgery, the prediction of the effective lens position (ELP) based on central corneal power measurements is susceptible to errors. Prior myopic corneal refractive surgery reduces the corneal refractive power and may result in a false prediction of a shallower ELP and a hyperopic surprise, due to underestimation of the IOL power 19 . It is therefore suggested to use as many approaches as possible to determine the IOL power selected based on the consensus of various methods 18 .

Fourth-generation IOL power calculation formulas use both corneal and IOL characteristics to improve the precision of optical calculations, unlike third-generation formulas which ignore the optical impacts of lens thickness and shape 11 . In this study, the Haigis-TK formula was used for IOL power calculations using the IOL Master 700 as it does not rely on keratometry measurements to calculate the ELP, improving IOL power calculations 20 , 21 . The ray-tracing technology used by the MS-39 device allows precise IOL power calculation by measuring all light rays at varying radial distances from the anterior and posterior corneal surfaces 22 .

The lack of a single established and reliable method for IOL power calculation in patients with prior corneal refractive surgery underscores the importance of this study. There are available novel no-history machine learning-based formulas, such as the PEARL-DGS, that represent an innovative approach to IOL calculation in post-refractive surgery eyes 23 . Although preliminary clinical validation studies have shown promising results, these machine learning-based formulas require extensive external validation and regulatory approval prior to their application in routine clinical practice 24 . In order to improve refractive outcome, intraoperative aberrometry has been proposed as a final verification tool of IOL power just before IOL implantation, though its use is limited by cost and accessibility 20 .

This study has several limitations that should be acknowledged. First, both eyes from some patients were included in the analysis, which may have introduced inter-eye correlation and reduced the statistical independence of the data. Second, the retrospective design limited control over measurement conditions and patient selection, potentially introducing bias. Third, postoperative refractive outcomes were not analysed, preventing direct validation of predictive accuracy against actual surgical results. Finally, the sample size, while sufficient for detecting inter-device differences, may not capture the full variability seen in the broader population of post-LASIK cataract patients.

In conclusion, in post-myopic LASIK eyes, accurate biometric evaluation remains essential yet challenging due to corneal surface modifications and altered anterior–posterior curvature ratios. This study demonstrated that, although the IOL Master 700 and the MS-39 produced statistically significant differences in several biometric parameters, these discrepancies did not result in clinically meaningful differences in emmetropic IOL power calculation or predicted postoperative refraction.

Finally, the strong agreement between devices in final refractive prediction suggests that both technologies are reliable tools for preoperative biometry in this complex patient population. Nevertheless, the differences observed in corneal parameters highlight the importance of understanding each deviceʼs measurement principles, particularly regarding anterior and posterior corneal surface assessment, to optimise IOL power selection. Using complementary data from multiple instruments may further enhance surgeon confidence and reduce refractive surprises in post-LASIK cataract surgery planning. Future prospective studies with larger and independent cohorts, and postoperative refractive outcome validation are warranted to confirm these findings and refine IOL power calculation strategies for surgically modified corneas.
